# Variations in *Quercus* sp. pollen seasons (1996–2011) in Poznań, Poland, in relation to meteorological parameters

**DOI:** 10.1007/s10453-013-9313-3

**Published:** 2013-08-07

**Authors:** Łukasz Grewling, Bogdan Jackowiak, Matt Smith

**Affiliations:** 1 Laboratory of Aeropalynology, Faculty of Biology, Adam Mickiewicz University, Umultowska 89, 61-614 Poznań, Poland; 2 Research Group Aerobiology and Pollen Information, Department of Oto-Rhino-Laryngology, Medical University of Vienna, Vienna, Austria

**Keywords:** Trends, Climate change, Central Europe

## Abstract

The aim of this study is to supply detailed information about oak (*Quercus* sp.) pollen seasons in Poznań, Poland, based on a 16-year aerobiological data series (1996–2011). The pollen data were collected using a volumetric spore trap of the Hirst design located in Poznań city center. The limits of the pollen seasons were calculated using the 95 % method. The influence of meteorological parameters on temporal variations in airborne pollen was examined using correlation analysis. Start and end dates of oak pollen seasons in Poznań varied markedly from year-to-year (14 and 17 days, respectively). Most of the pollen grains (around 75 % of the seasonal pollen index) were recorded within the first 2 weeks of the pollen season. The tenfold variation was observed between the least and the most intensive pollen seasons. These fluctuations were significantly related to the variation in the sum of rain during the period second fortnight of March to first fortnight of April the year before pollination (*r* = 0.799; *p* < 0.001). During the analyzing period, a significant advance in oak pollen season start dates was observed (−0.55 day/year; *p* = 0.021), which was linked with an increase in the mean temperature during the second half of March and first half of April (+0.2 °C; *p* = 0.014). Daily average oak pollen counts correlated positively with mean and maximum daily temperatures, and negatively with daily rainfall and daily mean relative humidity.

## Introduction

Oak (*Quercus* sp.) is of economic importance and as such is one of the most desirable broad-leaved trees in Polish forests (Boratyńska et al. 2006). Three native species of oaks, i.e., pedunculate oak (*Q. robur*), sessile oak (*Q. petraea*) and downy oak (*Q. pubescens*), occur in Poland. However, the distribution of the latter is limited to an isolated stand near to the Oder River (Zając and Zając 2001). *Q. robur* grows mainly in the oak-hornbeam forests on fertile loam soil whereas *Q. petraea* occupied more thermophilous and acidophilus oakwoods (Matuszkiewicz 2011). The most common species occurring in green spaces in Poznań is pedunculate oak (Jackowiak 1993). Apart from three native *Quercus* species, red oak (*Q. rubra*) was introduced to Poland from North America and is currently widespread in forest ecosystems (Woziwoda et al. 2012).

Oaks are anemophilous trees that produce enormous amount of pollen (Jato et al. 2007; Gómez-Casero et al. 2004; Tormo-Molina et al. 1996). Their staminate inflorescences are initiated in late spring 1 year before pollination, and the meiosis occurs in the following spring (Ducousso et al. 1993). Male flowers of *Quercus* species are evenly distributed on the long pendulous, catkin like inflorescences, and their mean number varies, depending on species, from 19 to 26 (for *Q. robur* and *Q. petraea*, respectively) (Chałupka 2006). For a given tree, catkin growth is achieved 1–2 weeks after bud opening and pollination is completed in 2–4 days. However, the rate of development depends on weather conditions (Ducousso et al. 1993). In Central and Western Europe, the flowering time is mainly limited to the April–May period (Corden and Millington 1999; Weryszko-Chmielewska et al. 2006; Kasprzyk 2009).

In general, oak pollen is not considered to be a potent aeroallergen, although the relationship between clinical and aerobiological data in the UK suggests that oak pollen could be an important cause of hay fever symptoms (Ross et al. 1996). This may be related to the high level of immunologic similarities between pollen of trees belonging to the Fagales order, e.g., hazel, alder, birch and oak (Wallner et al. 2009). A recent study revealed oak pollen contains allergens that cross-react with the birch pollen allergens Bet v 1, Bet v 2 and Bet v 4 (Egger et al. 2008). In addition, positive skin reactions to oak pollen allergens have been reported in many regions (Basak et al. 2011; Larenas-Linnemann et al. 2011; Kim and Cho 2012; García-Mozo et al. 1999). Results obtained in Warsaw, Poland (Rapiejko et al. 2004), showed that 19 % of allergic patients had positive skin reactions to *Quercus* pollen and the first allergic reaction in the patients with positive SPT occurred when daily average oak pollen levels reached 80 P m^−3^, a value that can be reached almost every year in Poznań (Stach 2006).

The behavior and course of *Quercus* pollen seasons have been comprehensively studied in Western Europe, especially in the UK and Spain (Corden and Millington 1999; García-Mozo et al. 2002; Jato et al. 2002; García-Mozo et al. 2008). However, there is still a dearth of data concerning the environmental factors affecting the production and release of *Quercus* pollen in Central Europe (Weryszko-Chmielewska et al. 2006; Kasprzyk 2009). This study supplies detailed information about the variations, trends and climatic requirements of oak pollen seasons in Poznań, Poland.

## Methods

### Study area and climate

Poznań, population ~550,000, is the capital of Wielkopolska, an agricultural region located in mid-western Poland (CSO 2011). Poznań has a temperate continental climate with cold winters and warm summers. January is the coldest month of the year with the mean monthly temperature −1.6 °C, whereas July is the warmest one (18.1 °C). The mean annual precipitation is 517 mm (1951–2000 average). Westerly winds predominate, particularly from the SW (Woś 2010).

### Aerobiological data

Quercus pollen data (1996–2011) were collected in Poznań by volumetric spore trap of the Hirst design (Hirst 1952). The trap was situated 33 m above ground level, about 1 km southwest of the city center (52°24′N 16°53′E). In the neighborhood around the pollen-monitoring site, there are 4–8 storey buildings, two small parks, gardens, patches of grass and ruderal vegetation. The nearest oak stand with 64 trees of *Q. robur* and 7 of *Q. rubra* is located in Manitius Park approximately 500 m south from the pollen trap.

Two different pollen-counting methods have been employed. From 1996 to 1999, pollen data were collected following the methods outlined by Stach (2000) where pollen grains were counted along twelve latitudinal transects. From 2000 to 2011, this method was changed and pollen grains were counted along four longitudinal transects, which were divided into 2-mm (1 hourly) intervals, following the method described by the Spanish Aerobiological Network (Galán et al. 2007). The two *Quercus* pollen datasets from Poznań were spliced together to make a single dataset running from 1996 to 2011. Both counting methods have been shown to produce comparable results (Cariñanos et al. 2000). Daily average and bi-hourly concentrations of pollen grains are expressed as P m^−3^ (Comtois 1998).

The limits of the oak pollen season were calculated by using the 95 % method (Nilsson and Persson 1981) whereby the season starts when 2.5 % of the total catch was achieved and ends when 97.5 % is reached. Data related to start, end and peak day were converted to the day of the year from 1 January (DOY). Due to the trap failure during the 2008 oak pollen season, the dataset contained missing values, which were replaced by the mean daily average *Quercus* pollen count for that day (1996–2001 mean): 3 days, 9–11 May 2008.

The following characteristics of the oak pollen season were examined: start date, end date, duration (number of days), the duration of the pre-peak period (number of days from the start of the oak pollen season to the peak date), the intensity of the oak pollen season (season pollen index, SPI), the timing and magnitude of the peak day (the highest daily average pollen concentration during pollen season), percentage of the total SPI recorded during 4 × 1 weekly periods during the oak pollen season (%) and the number of days during the season with daily average oak pollen levels >80 P m^−3^. This threshold value (80 P m^−3^) was based on atmospheric concentrations of oak pollen reported to evoke allergic symptoms in Poland (Rapiejko et al. 2004).

### Meteorological data

Meteorological data, i.e., daily average maximum, minimum and mean temperature, daily rainfall and relative humidity (RH), were obtained from the station located at Ławica Airport (52°25′N 16°49′E) approximately 7 km from the city center and 4.25 km west of the pollen-monitoring site. In order to analyze the influence of weather conditions on variations and trends (expressed by linear function, data not shown) of oak pollen seasons, the following meteorological parameters were examined:

- Fortnightly, monthly, 1.5-monthly and 2-monthly mean, maximum and minimum daily average temperatures and daily rainfall from the year preceding pollination (1995–2010), as well as from the same year as pollination (1996–2011, January–April);
- Averages of daily mean, maximum and minimum temperatures and daily rainfall recorded during the first and second week of oak pollen season and during the whole pollen season.

### Statistical analysis

Correlations between chosen meteorological parameters (mentioned in Sect. 2.3) and particular characteristics of *Quercus* pollen season (mentioned in Sect. 2.2) were examined using the parametric Pearson correlation test. The relationship between daily average oak pollen concentrations and the daily meteorological data (maximum, minimum and mean temperature, daily rainfall and RH) was examined using nonparametric Spearman correlation analysis. Parametric (Pearson) or nonparametric (Spearman) analysis was applied depending on data distribution (checked by Shapiro–Wilk test with *p* level <0.05, results not shown). Simple linear regression analysis was used to describe trends of the chosen characteristics of the oak pollen season. The following statistics are shown: the slope of the regression (β); coefficient of determination (*R* ^2^); and probability level (*p*). All calculations were carried out using software Microsoft Excel, XLSTAT 2010 and Statistica 10.

## Results

Start and end dates of oak pollen seasons in Poznań during the studied period varied by about 2 weeks annually (Table 1). In 2006 and 2010, the onset of the oak pollen season was observed during the third week of April (111 DOY), whereas in 1997, the oak pollen season started at the beginning of May (125 DOY). Usually the oak pollen grains were recorded in the air through the whole of May, with the highest daily pollen concentrations occurring in the first week of the season. On peak days, the mean daily average oak pollen level exceeded 210 P m^−3^. Almost 75 % of the total SPI was recorded within the first 2 weeks of the pollen season. However, this value varied greatly depending on year (from 8.1 % in 2006 to 93.2 % in 2009). Tenfold differences were observed between the least and the most intensive pollen seasons (229 grains in 1997 and 2,514 grains in 2009). During the latter, high daily average pollen concentrations able to induce allergy reactions (>80 P m^−3^) were recorded on 9 days.

**Table 1:** Characteristics and trends of oak pollen seasons in Poznań (1996–2011)

Years	Start (DOY)	End (DOY)	Duration (days)	SPI (grains)	Peak value (P/m^3^)	Peak day (DOY)	Duration of pre-peak period (days)	Numbers of days with pollen level above 80 P/m^3^	Percentage of total SPI during 4 × 1 weekly periods of pollen season			
									1st week	2nd week	3rd week	4th week
1996	124	150	27	1996	512	134	11	7	28.3	53.2	15.9	3.1
1997	125	158	34	229	48	137	13	0	12.2	62.9	15.3	5.7
1998	118	143	26	2037	298	129	12	8	29.6	54.8	8.8	7.9
1999	114	140	27	658	105	120	7	2	11.4	64.4	15.3	10.3
2000	115	139	25	672	123	119	5	1	59.7	29.9	7.4	3.1
2001	121	150	30	972	130	131	11	5	20.6	60.2	14.7	3.6
2002	120	155	36	1291	211	124	5	5	57.4	14.6	27.7	0.6
2003	120	153	34	1500	260	125	6	7	67.6	22.1	6.9	2.5
2004	120	157	38	586	94	125	6	2	59.0	21.2	10.9	5.3
2005	121	159	39	809	84	123	3	1	32.8	16.9	29.0	16.2
2006	111	148	38	836	145	134	24	2	7.5	0.6	26.7	53.9
2007	113	138	26	999	169	125	13	2	35.3	55.6	7.8	2.1
2008	122	147	26	1248	249	133	12	2	19.2	43.1	12.8	5.8
2009	113	132	20	2514	424	116	4	9	52.1	41.1	7.4	1.4
2010	111	150	40	1479	296	120	10	5	1.2	49.0	23.9	14.3
2011	114	136	23	1570	214	117	4	6	59.0	26.2	13.9	1.8
Mean	118	147	31	1212	210	126	9	4	35	38	15	9
Min	111.0	132.0	20.0	229.0	48.0	116.0	3.0	0.0	1.2	0.6	6.9	0.6
Max	125.0	159.0	40.0	2514.0	512.0	137.0	24.0	9.0	67.6	64.4	29.0	53.9
β	**−0**.**553**	−0.549	0.004	28.921	0.794	−0.659	−0.106	0.053	0.469	−1.529	0.169	0.405
R^2^	**0**.**327**	0.098	0.000	0.050	0.005	0.227	0.009	0.008	0.010	0.134	0.011	0.022
p	**0**.**021**	0.239	0.990	0.403	1.891	0.062	0.727	0.743	0.706	0.162	0.696	0.581

**Fig. 1:**
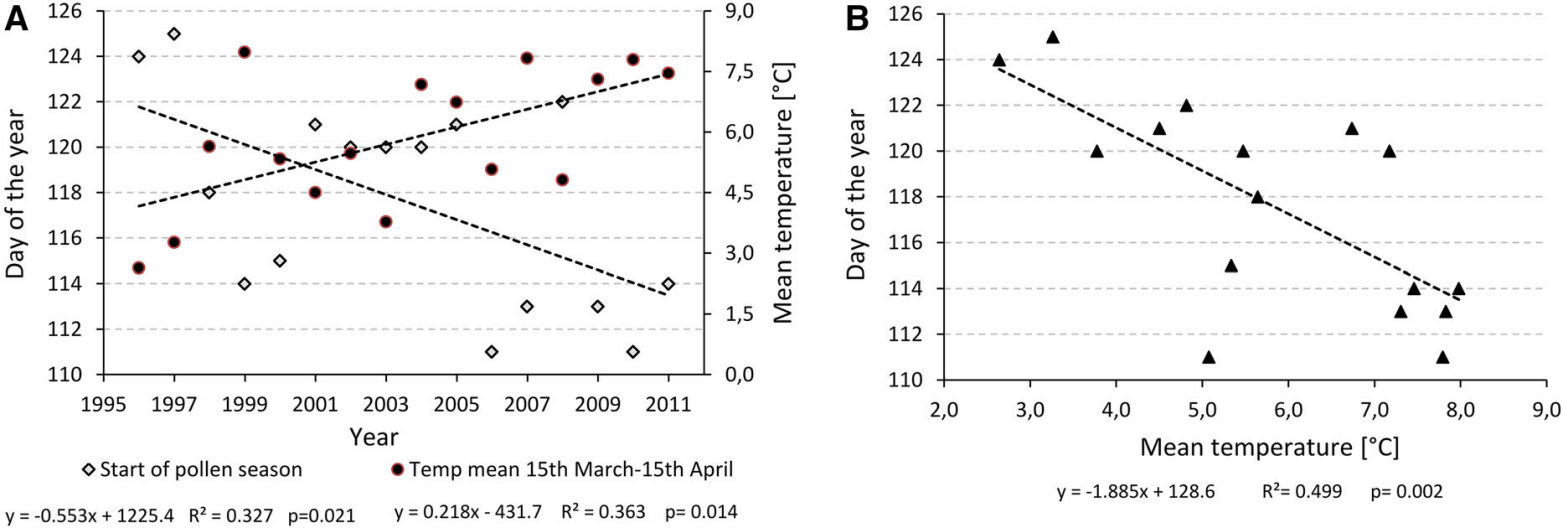
Trends in oak pollen season start dates and daily mean temperature during the 15 March–15 April (**A**) and graphical presentation of the relationship between these two parameters (**B**)

Only one of the analyzed characteristics of oak pollen seasons had a significant trend during the 16-year period of pollen monitoring (Table 1; Fig. 1a). Start dates of oak pollen seasons in Poznań advanced −0.55 day/year (*p* = 0.021).

Correlation analysis showed a number of statistically significant (*p* < 0.05) relationships between the timing of oak pollen seasons in Poznań (i.e., start, end and peak dates) and meteorological data recorded before the start of the oak pollen season (in March and April) (Table 2). The mean temperature during the second fortnight of March and first fortnight of April markedly influenced the onset of the pollen season (*r* = −0.707; *p* < 0.01) and peak date (*r* = −0.753; *p* < 0.001), whereas the end date of the pollen season was significantly affected by the mean April temperature (*r* = −0.789; *p* < 0.001).

**Table 2:** Selected significant Pearson correlations coefficients between particular characteristics of oak pollen seasons and meteorological parameters (576 correlations in total/75 significant)

Pollen season parameter	Period	Meteorological parameter	Pearson coefficient
*Meteorological parameters recorded during the same year as pollen season*			
Pollen season start	2nd fortnight of March–1st fortnight of April	Temp mean (°C)	−0.707**
	2nd fortnight of March–1st fortnight of April	Temp min (°C)	−0.686**
	2nd fortnight of March–1st fortnight of April	Temp max (°C)	−0.668**
	2nd fortnight of March	Temp min (°C)	−0.641**
	2nd fortnight of March	Temp mean (°C)	−0.639**
	2nd fortnight of March	Rainfall (mm)	−0.634**
	1st fortnight of April	Temp mean (°C)	−0.590*
Pollen season end	April	Temp mean (°C)	−0.789***
	April	Temp max (°C)	−0.742**
	April	Temp min (°C)	−0.714**
	1st fortnight of April	Temp min (°C)	−0.610*
	1st fortnight of April	Temp mean (°C)	−0.595*
	2nd fortnight of April	Temp max (°C)	−0.574*
	2nd fortnight of March–1st fortnight of April	Temp mean (°C)	−0.564*
Peak day	2nd fortnight of March–1st fortnight of April	Temp max (°C)	−0.819***
	2nd fortnight of March–1st fortnight of April	Temp mean (°C)	−0.753***
	1st fortnight of April	Temp max (°C)	−0.693**
	1st fortnight of April	Temp mean (°C)	−0.683**
	2nd fortnight of March	Temp max (°C)	−0.634*
	2nd fortnight of March	Temp mean (°C)	−0.604*
	2nd fortnight of March–1st fortnight of April	Temp min (°C)	−0.592*
*Meteorological parameters recorded 1* *year before pollen season*			
SPI	2nd fortnight of March–1st fortnight of April	Rainfall (mm)	0.799***
	2nd fortnight of March–April	Rainfall (mm)	0.763***
	February–1st fortnight of March	Temp mean (°C)	0.643**
	February–1st fortnight of March	Temp min (°C)	0.625*
	February	Temp mean (°C)	0.619*
	February	Temp min (°C)	0.617*
	August–2nd fortnight of September	Rainfall (mm)	0.556*

* *p* < 0.05; ** *p* < 0.01; *** *p* < 0.001

No significant relationships were observed between SPI and meteorological data recorded during the same year as pollination. However, several significant correlations were detected between SPI and the meteorological parameters recorded the previous year. For instance, the amount of rainfall during the period second fortnight of March to first fortnight of April as well as during the period August to the second fortnight of September the year before pollination positively correlated with the pollen season intensity (*r* = 0.799; *p* < 0.001 and *r* = 0.556; *p* < 0.05, respectively).

In addition, the analysis revealed that the weather conditions during the first 2 weeks of the oak pollen season significantly influenced the course of the season as a whole (Table 3). The length of the oak pollen season positively correlated with the amount of rain recorded during the second week of the season (*r* = 0.719; *p* < 0.01). Also, the number of oak pollen collected in the first week of the oak pollen season (expressed in % of SPI) depended significantly (*r* = 0.792; *p* < 0.001) on the mean temperature recorded during that period.

**Table 3:** Selected significant Pearson correlations coefficients between the characteristics of oak pollen season and meteorological parameters recorded during the pollen season (60 correlations in total/11 significant)

Pollen season parameter	Meteorological parameters	Correlation coefficient
Percentage of pollen during first week of pollen season	Temp mean (°C) during first week of pollen season	0.792***
	Temp max (°C) during first week of pollen season	0.736**
	Temp min (°C) during first week of pollen season	0.693**
Length of pollen season	Sum of rain (mm) during second week of pollen season	0.719**
	Sum of rain (mm) during whole pollen season	0.690**

* *p* < 0.05; ** *p* < 0.01; *** *p* < 0.001

Daily variations in oak pollen concentrations were strongly influenced by the meteorological parameters measured on the same day (Table 4). Daily average oak pollen counts correlated positively with mean and maximum daily temperatures, and negatively with daily rainfall and daily mean relative humidity.

**Table 4:** Spearman correlation coefficients between daily oak pollen concentrations and selected meteorological parameters

Years	Temp mean (°C)	Temp max (°C)	Temp min (°C)	Rainfall (mm)	Humidity (%)
1996	ns	ns	ns	ns	ns
1997	0.507**	0.538**	0.441*	ns	ns
1998	0.500*	0.435*	0.583**	ns	ns
1999	ns	ns	−0.506**	−0.413*	−0.490*
2000	ns	ns	0.421*	ns	ns
2001	ns	ns	ns	ns	ns
2002	ns	ns	ns	ns	ns
2003	ns	ns	ns	ns	ns
2004	ns	ns	ns	ns	ns
2005	ns	ns	ns	ns	ns
2006	0.505**	0.466**	ns	ns	−0.406*
2007	ns	ns	ns	−0.548**	−0.814***
2008	ns	0.461*	ns	ns	−0.622***
2009	0.575**	0.686**	ns	−0.707***	−0.698***
2010	0.328*	ns	ns	−0.327*	ns
2011	ns	ns	ns	−0.587**	−0.691***
1996–2011	0.170***	0.204***	ns	−0.289***	−0.286***

*ns* not significant

* *p* < 0.05; ** *p* < 0.01; *** *p* < 0.001

## Discussion

This study shows a clear relationship (*r* = −0.707; *p* < 0.01) between start dates of *Quercus* pollen seasons recorded in Poznań and spring temperatures, i.e., mean temperature from the second half of March to the first half of April. These results concur with the findings of previous studies that have identified significant negative correlations between the start dates of tree pollen seasons and temperatures recorded just few weeks before pollination (Frei and Gassner 2008; Rodríguez-Rajo et al. 2009; Grewling et al. 2012). In the case of oak pollen seasons in Poznań, we have shown that start dates occur 1.9 days earlier with every 1.0 °C increase in temperature. Interestingly, during the analyzed period (1996–2011), a significant advance of *Quercus* pollen season start dates was noticed (−0.55 day/year; *p* = 0.021), which supports reported global tendencies to earlier development of many plants during the last decades due to climatic warming (Fujisawa and Kobayashi 2010; García-Mozo et al. 2010; Grab and Craparo 2011; Ma and Zhou 2012). The recorded shift in the timing of oak pollen seasons is likely to be related to significant increases (+0.2 °C/year; *p* = 0.014) in mean daily temperatures between the second fortnight of March and first fortnight of April in Poznań, which is the crucial parameter for oak catkins development (Fig. 1b).

Such clear relationships with weather conditions were not observed with reference to the seasonal sum of *Quercus* pollen. Statistical analysis between the SPI of oak and meteorological parameters recorded during the year of pollination in Poznań did not reveal any significant correlations. This is at odds with previously reported results from Galicia, the northwest part of Spain (García-Mozo et al. 2006). It should be noted that in this region of Iberian Peninsula, as in Poland, the most common oak species is *Q. robur* (Rodríguez-Rajo et al. 2005). The observed divergence in obtained results probably reflects different adaptation of this species to local climatic conditions. On the other hand, Andersen (1980) and Corden and Millington (1999) showed that intensive oak pollen seasons in Western Europe are related to the high temperatures in summer months during previous growing seasons. Similarly, Emberlin et al. (1990) linked sunny, warm autumn period with the high pollen production of many tree species the following year, including *Quercus*. Based on 16 years of aerobiological data, such relations were not observed in Poznań. In turn, our study revealed significant correlations between the SPI of oak and the sum of rain in the second half of March and first half of April in the year prior to flowering (*r* = 0.799, *p* < 0.001). Although the casual mechanism of this relationship is not clear, the above mentioned meteorological parameters might influence the first phases of the oak microsporogenesis that occurs in the spring 1 year before flowering (Ducousso et al. 1993) and thereby affects the number of pollen grains produced.

The analysis of the pollen season intensity is hindered by the fact that several oak species occur in Poznań and its surroundings. Despite the fact that the most common oak species in the city is *Q. robur*, the other *Quercus* species (e.g., *Q. petraea* and *Q. rubra*) are well represented in the forests near to Poznań (Krotoska 1961; Żukowski et al. 2005). In addition, almost 20 different oak species are grown in the University Botanical Garden in Poznań (about 1.5 km from the pollen-monitoring site), although many of them do not exceed the generative stage and therefore do not produce pollen (Węglarska and Węglarski 2007). Taking into account that the different oak species have different ecological requirements (Timbal and Aussenac 1996; Bugała 2006), it is reasonable to assume that the weather conditions do not have the same influence on the pollen production of different oak species. The pollen grains are morphologically similar, and so it is not possible to distinguish them under light microscope. As a result, all collected oak pollen grains were gathered together and classified on genus level in the present study. Thus, the results of statistical analysis of aerobiological data, especially intensity of pollen seasons, can be biased and should be examined carefully.

The mean SPI value, which in Poznań exceeded 1212 grains during the studied period, was similar to those observed in other sites in Poland (Weryszko-Chmielewska et al. 2006) as well as in Western Europe, e.g., in the UK (Corden and Millington 1999), Belgium and the Netherland (Spieksma et al. 2003). Also in Galicia (NW Spain), the intensity of *Quercus* pollen seasons is comparable to Poznań (García-Mozo et al. 2006). The concentration of oak pollen is, however, much higher in southern France and central Spain (Skjøth et al. 2013) which is likely to be related to the higher number of *Quercus* species and wider distribution of oak trees in those areas (García-Mozo et al. 2006; Skjøth et al. 2008). General trends toward increasing seasonal sums of pollen from allergenic plants have been reported in Europe (Ziello et al. 2012), but such a trend was not seen in relation to *Quercus* in Poznań. This could again express the local response of this taxa to microclimate conditions in the region or be related to such factors as the length of aerobiological pollen data series that were previously discussed for *Betula* pollen seasons in Poznań (Grewling et al. 2012). From the standpoint of sensitized patients, the large year-to year variation in SPI as well as observed advance of *Quercus* pollen season start dates should be considered in allergy prophylaxis. In addition, it is worth emphasizing that the daily oak pollen concentrations that evoke the first allergic symptoms to oak pollen, i.e., >80 P m^−3^ (Rapiejko et al. 2007), were often exceeded during peak days and lasted for more than 1 week. Such information would be important for both patients and allergists.

Similarly, one of the most examined characteristics of oak pollen seasons, duration, also varied markedly. For instance, in 2008, the oak pollen season ended after 20 days, whereas 1 year later, the season was twice as long. The extension of the pollen season was mainly caused by an increase in daily rainfall during its second week (*r* = 0.719; *p* < 0.01), which suggests that the release of oak pollen grains from catkins is strongly hindered by rain episodes. In addition, the negative influence of rain and RH on daily concentrations of oak pollen (*r* = −0.289; *p* < 0.001 and *r* = −0.286; *p* < 0.001, respectively) agrees with previous studies (Rodríguez-Rajo et al. 2005; Weryszko-Chmielewska et al. 2006; Rodríguez de la Cruz et al. 2008). In general, low relative humidity accelerates anther opening, while high relative humidity delays or inhibits the process (Carrizo García et al. 2006).

From a physiological point of view, the opening of the anther is preceded by rarefaction of locular fluid between pollen grains, which might be caused by reabsorption of fluid through anther tissue or by evaporation. Reabsorption is programmed and regulated by the plant, whereas evaporation depends on external factors, like ambient RH, temperature and wind speed (Pacini and Hesse 2004; Dahl et al. 2013). In the majority of a plant’s anther, opening is considered as a unidirectional process—once opened anthers are not able to close again and pollen grains are released from the anther as soon as it opens (Pacini 2000). On the other hand, anthers of some species can close in response to varying RH (and later re-opened again), which is likely to prevent hydration of the pollen (Edwards and Jordan 1992; Li et al. 2012; Dahl et al. 2013). Discontinuous pollen liberation due to reversible anther opening allows unfavorable conditions during pollination to be avoided and enhances pollen competence, thus making the plant better adapted to rainy flowering seasons (Li et al. 2012). However, this phenomenon was only described in entomophilous plants (Edwards and Jordan 1992; Wang et al. 2009; Li et al. 2012), and there is a lack of evidences whether it occurs in wind-pollinated species, such as oak. Nevertheless, this mechanism could at least partially explain how the adverse weather conditions affect the duration of pollen seasons.

## Conclusions

This study supplies important information about the environmental factors that effect *Quercus* pollen seasons. Such information may have further applications in different scientific areas like plant physiology research, forest ecology, biometeorology and allergological prophylaxis. The significant pollen-weather relationships could also be incorporated into models predicting the impacts of future climate change on the course of oak pollen seasons in Central Europe. One of the most notable results of this study was the significant advance of *Quercus* pollen seasons start dates (−0.55 day/year; *p* = 0.021) that may be linked with recent increases in the daily mean temperatures one month before pollination (+0.2 °C/year; *p* = 0.014). The other characteristics of oak pollen seasons did not reveal such distinct trends. Year-to-year fluctuations in the intensity of oak pollen seasons can be partly explained by rainfall during the first phases of oak microsporogenesis (the year before pollination) that could influence pollen development. In addition, the duration of the oak pollen season can be extended by rainfall hindering pollen release, especially during the second week of pollen season. However, to have the clear picture of observed mechanisms, more detailed investigations are desirable, especially those focused on examining the physiological basis of the anther opening behavior and pollen liberation in *Quercus* species.
